# FOXL2 and DMRT1L Are Yin and Yang Genes for Determining Timing of Sex Differentiation in the Bivalve Mollusk *Patinopecten yessoensis*

**DOI:** 10.3389/fphys.2018.01166

**Published:** 2018-08-22

**Authors:** Ruojiao Li, Lingling Zhang, Wanru Li, Yang Zhang, Yangping Li, Meiwei Zhang, Liang Zhao, Xiaoli Hu, Shi Wang, Zhenmin Bao

**Affiliations:** ^1^MOE Key Laboratory of Marine Genetics and Breeding, Ocean University of China, Qingdao, China; ^2^Laboratory for Marine Biology and Biotechnology, Qingdao National Laboratory for Marine Science and Technology, Qingdao, China; ^3^Laboratory for Marine Fisheries Science and Food Production Processes, Qingdao National Laboratory for Marine Science and Technology, Qingdao, China

**Keywords:** Yesso scallop, sex differentiation, FOXL2, DMRT1L, LOG_10_(*DMRT1L*/*FOXL2*)

## Abstract

Sex determination and differentiation have long been a research hotspot in metazoans. However, little is known about when and how sex differentiation occurs in most mollusks. In this study, we conducted a combined morphological and molecular study on sex differentiation in the Yesso scallop *Patinopecten yessoensis*. Histological examination on gonads from 5- to 13-month-old juveniles revealed that the morphological sex differentiation occurred at 10 months of age. To determine the onset of molecular sex differentiation, molecular markers were screened for early identification of sex. The gonadal expression profiles of eight candidate genes for sex determination or differentiation showed that only two genes displayed sexually dimorphic expression, with *FOXL2* being abundant in ovaries and *DMRT1L* in testes. *In situ* hybridization revealed that both of them were detected in germ cells and follicle cells. We therefore developed LOG_10_(*DMRT1L*/*FOXL2*) for scallop sex identification and confirmed its feasibility in differentiated individuals. By tracing its changes in 5- to 13-month-old juveniles, molecular sex differentiation time was determined: some scallops differentiate early in September when they are 7 months old, and some do late in December when they are 10 months old. Two kinds of coexpression patterns were found between *FOXL2* and *DMRT1L*: expected antagonism after differentiation and unexpected coordination before differentiation. Our results revealed that scallop sex differentiation co-occurs with the formation of follicles, and molecular sex differentiation is established prior to morphological sex differentiation. Our study will assist in a better understanding of the molecular mechanism underlying bivalve sex differentiation.

## Introduction

Sexual reproduction is one of the most universal phenomena that widely exist in animals. As a focus of this research area, sexual development encompasses sex determination and differentiation. The former is defined as the process by which sex is established under the influence of genetic or environmental factors and the latter as the process that an undifferentiated gonad uses to transform into an ovary or a testis (Bull, 1983; Penman and Piferrer, 2008; Piferrer and Guiguen, 2008). Therefore, sexual development is a complex network that is initiated by a sex-determining trigger mediating the expression of sex differentiation genes, which ultimately gives rise to the phenotypic differences between sexes (Heule et al., 2014).

Sex determination and differentiation have received much research attention in various species. For example, in mammals, genotypic sex is defined by the presence or absence of sex-specific chromosome Y that carries the dominant male determinant SRY (Swain and Lovell-Badge, 1999; Kocer et al., 2009). In chickens that possess a ZZ/ZW sex chromosome system, DMRT1 is required for testis determination (Zarkower, 2001; Smith et al., 2009). In *Caenorhabditis elegans* and *Drosophila melanogaster*, a cascade of sex switch genes is controlled by the ratio of X chromosomes to sets of autosomes (the X:A ratio; Zarkower, 2001; Goodwin and Ellis, 2002). It seems sex is determined by sex chromosomes and controlled by some master switch genes in many organisms.

Mollusca represents the second largest phylum of invertebrates after Arthropoda. Bivalves are a large group of mollusks that exhibit different reproductive strategies: some are gonochoric, some are hermaphroditic, and some are capable of sex changes. Although much research has been done on many different bivalves, there is no clear evidence for the existence of sex chromosomes in these animals. However, some progress has been made regarding the identification of sex determination or differentiation genes. Several key sex-related genes in model species have been characterized in bivalves, including FOXL2 (Naimi et al., 2009a; Liu et al., 2012), WNT4 (Li et al., 2013; Yang et al., 2015), FST (follistatin; Ni et al., 2012), β-catenin (Li et al., 2014b; Santerre et al., 2014), DMRT (Naimi et al., 2009b; Feng et al., 2010; Yu et al., 2011; Shi et al., 2014), DAX1 (Li et al., 2014a), and SOXE (Santerre et al., 2014). Many of them display sexually dimorphic expression patterns in the gonads and are regarded as participants in the sex differentiation cascade. Meanwhile, gonadal transcriptome analyses identified some key candidate genes for sex determination or differentiation, such as FOXL2, WNT4, β-catenin, DMRT, DAX1, SOXE, and SOXH (Teaniniuraitemoana et al., 2014; Zhang et al., 2014; Tong et al., 2015; Li et al., 2016; Patnaik et al., 2016). These studies suggest that sex determination and differentiation genes may be deeply conserved in animals.

Yesso scallop *Patinopecten yessoensis* is a commercially important species widely cultured in China and Japan. It is predominantly gonochoric, with scarce hermaphroditism. Due to its commercial importance, much work has been performed to obtain an understanding of its reproductive process (Osada et al., 2004; Tanabe et al., 2010; Nagasawa et al., 2015). Recently, our group completed whole genome sequencing and gonadal transcriptome analysis of *P. yessoensis* (Li et al., 2016; Wang et al., 2017), which provide valuable resources for unraveling the molecular mechanisms underlying scallop sex differentiation. In the present study, morphological and molecular sex differentiation was examined in the Yesso scallop. Eight candidate genes for sex determination or differentiation were chosen to establish markers for sex identification and the onset of molecular sex differentiation was determined; we also compared the molecular changes in the undifferentiated and differentiated gonads. This study will pave the way for a better understanding of the regulatory network in bivalve sex differentiation.

## Materials and Methods

### Sample Collection

To obtain adult scallops with known sexes, 15-month-old mature female and male individuals were selected in May 2015, cultured in separate cages, and transported to the laboratory every month from August 2015 to March 2016. Meanwhile, juvenile scallops 5–13 months of age were obtained from July 2015 to March 2016. All of these scallops were collected from the Dalian Zhangzidao Fishery Group Corporation (Liaoning Province, China) and acclimated in filtered and aerated seawater for 1 week at the temperature at which they were collected. For each month, gonads of 50 adults and/or 50 juveniles were dissected: the majority were immediately frozen in liquid nitrogen and stored at −80°C for RNA extraction, and the remainder were fixed in 4% paraformaldehyde overnight, dehydrated with serial methanol (25, 50, 75, and 100%) diluted in 0.01 M phosphate-buffered saline and then stored at −20°C for paraffin sectioning and *in situ* hybridization. Our experiments were conducted according to the guidelines and regulations established by the Ocean University of China and the local government.

### Histology

The samples were transferred to ethanol, cleared in xylene, embedded in paraffin wax, and cut into 5-μm-thick sections on a rotary microtome (Leica, Wetzlar, Germany). Serial sections were tiled on glass slides, deparaffinized with xylene, hydrated with graded ethanol to water, and stained with hematoxylin. After that, the glass slides were counterstained with eosin, dehydrated with ethanol, cleared with xylene, mounted with neutral balsam, and covered with coverslips. Finally, the sections were observed under a Nikon’s Eclipse E600 research microscope.

### Candidate Genes Identification

Four ovary-related genes (FOXL2, WNT4, FST, β-catenin) and four testis-related genes (DMRT1L, DAX1, SOXE, and SOXH) were selected for analysis. To identify these genes, protein sequences from other organisms were collected from NCBI and used as queries against the genome (Wang et al., 2017) and gonadal transcriptomes (Li et al., 2016) by TBLASTN with an e-value threshold of 1e^−5^. The resultant scallop sequences were further confirmed by BLASTX against the NCBI protein sequence database.

### Phylogenetic Analyses

Full-length protein sequences encoding FOXL2/3 and DMRT homologs were downloaded from NCBI. Multiple sequence alignments (**Supplementary Material**) were conducted using ClustalW2 program with default parameters (Larkin et al., 2007). The neighbor-joining phylogenetic trees were constructed using MEGA 6 (Tamura et al., 2013), with a Poisson model and assuming uniform rates among sites. Bootstrapping with 1000 replications was conducted to evaluate the phylogenetic tree.

### RNA Extraction

Total RNA was isolated using the conventional guanidinium isothiocyanate method and digested with DNase I (TaKaRa, Shiga, Japan) to remove potential DNA contamination. RNA concentration and purity were determined by Nanovue Plus spectrophotometer (GE Healthcare, Piscataway, NJ, United States), and RNA integrity was verified by agarose gel electrophoresis. Only RNA samples with clear bands corresponding to 18S and 28S rRNA on the gel, an OD260/OD280 ratio between 1.8–2.0, and an OD260/OD230 ratio higher than 2.0 were used for subsequent experiments.

### Reverse Transcription Quantitative-PCR (RT-qPCR)

First-strand cDNA was synthesized from 2 μg total RNA using oligo(dT)_18_ and MMLV reverse transcriptase (TaKaRa, Shiga, Japan) in a volume of 20 μl. The reaction was performed at 42°C for 90 min and terminated by heating at 70°C for 10 min. Finally, the cDNA products were diluted to 10 ng/μl and stored at −20°C. Gene-specific primers were designed using Primer Premier 5.0 and listed in **Table 1**. Amplification efficiency of each primer pair was calculated based on the standard curve generated from a twofold dilution series spanning six orders of magnitude. Quantitative-PCR was conducted using Light Cycler 480 SYBR Green I Master on a Light Cycler 480 Real-time PCR System (Roche Diagnostics, Mannheim, Germany) with the following program: 94°C for 10 min, followed by 40 cycles of 94°C for 15 s and 60°C for 1 min. For each month, six to eight samples were assayed and all reactions were conducted in triplicate. To ensure that the RT-qPCR ran properly, negative controls, including NTCs (no-template controls) and no-reverse transcription controls, and positive controls were included in each run. Melting curve analysis was performed to verify that each primer set amplified a single product (**Supplementary Figure S1**). *EF1A* (elongation factor 1-alpha), which was stably expressed throughout the entire experiment, was used as an endogenous control for the normalization of gene expression (Santerre et al., 2013; Teaniniuraitemoana et al., 2015; Li et al., 2016). The relative expression level of each gene was calculated using the 2^−ΔΔCt^ method. Statistical analysis was performed by Welch’s *t*-tests. Pearson’s correlation coefficient was calculated to explore the relationships between the two genes. *P*-values lower than 0.05 were considered statistically significant.

**Table 1:** Sequences of all primers used for RT-qPCR.

Gene name	Primer sequences (5′–3′)	Amplicon length (bp)	Amplification efficiency	GenBank Acc. No
EF1A	F:CCATCTGCTCTGACAACTGA	196	1.02	XM_021500266.1
	R:GGACAATAACCTGAGCCATAA			
FOXL2	F:AACTTCTGGACATTGGACCCTGCTT	134	1.00	XM_021497746.1
	R:CCGCAGTGGTTGTCAGCAAATAAGG			
WNT4	F:ATGAATAGCGTGGCAGCAAT	141	0.97	XM_021498826.1
	R:ACTCGTCTATAGCCGAATGA			
FST	F:CCAATCCTAACTTCGTGTGT	105	1.04	XM_021490283.1
	R:CCATAGGCGATACGTATTGA			
β-catenin	F:GCAACACCAGGATGATGAAT	112	1.03	XM_021507890.1
	R:ATCCTGCATGTAGGTGTTCT			
DMRT1L	F:ACAGATTCCCTACAGATGCT	128	0.98	XM_021498039.1
	R:TTATTCATGGCGGCGTCTAT			
DAX1	F:CGTGTCCTACAACAGTAACA	117	0.98	XM_021500761.1
	R:GTGGTCCATTGCTACCTTAT			
SOXE	F:CTCTGGAGGCTTCTGAATGA	144	1.01	XM_021493168.1
	R:TCTGTCCTCCACTAGCACTT			
SOXH	F:CATGCCTGGTACCTCTATGA	172	0.99	XM_021485311.1
	R:GGCCGAGTCGAACACTGATT			

### *In situ* Hybridization

To prevent cross-detection of other FOX and DMRT genes, cDNA fragments avoiding conserved DNA binding domains were amplified with specific primers (**Table 2**) containing a 5′ T7 promoter sequence (5′-TAATACGACTCACTATAGGG-3′). Purified PCR products were used as templates for *in vitro* transcription. Digoxigenin-labeled sense and anti-sense probes were generated using the DIG RNA Labeling Mix (Roche, Mannheim, Germany) and T7 RNA polymerase (Thermo, Waltham, MA, United States). Sections of the gonadal tissues were serially rehydrated in PBST (phosphate-buffered saline plus 0.1% Tween-20) and digested with 2 μg/ml proteinase K at 37°C for 15 min. After pre-hybridization at 60°C for 4 h, hybridization was performed with 1 μg/ml denatured RNA probe in hybridization buffer (50% formamide, 5× SSC, 100 μg/ml yeast tRNA, 1.5% blocking reagent, 0.1% Tween-20) at 60°C for 16 h. Then, the probes were washed away, and antibody incubation was performed in a fresh solution of anti-digoxigenin-AP Fab fragments (Roche, Mannheim, Germany) coupled with blocking buffer (diluted 1:2000) at 4°C for 16 h. After extensive washing with maleic acid buffer (0.1 M maleic acid, 0.15 M NaCl, 0.1% Tween-20, pH = 7.5), sections were incubated with nitro blue tetrazolium/5-bromo-4-chloro-3-indolyl phosphate (NBT/BCIP) substrate solution and counterstained with 1% neutral red solution.

**Table 2:** Sequences of primers used for *in situ* hybridization.

Gene name	Primer sequences (5′–3′)
FOXL2	F:CTTATTTGCTGACAACCACTGCG
	R:TAGGGGCCGAACGGAAAGG
	F-T7:TAATACGACTCACTATAGGGCTTATTTGCTGACAACCACTGCG
	R-T 7:TAATACGACTCACTATAGGGTAGGGGCCGAACGGAAAGG
DMRT1L	F:GGACACCATCACGCATACCAA
	R:ACCAGAGTTCCTTCCGCCTC
	F-T7:TAATACGACTCACTATAGGGGGACACCATCACGCATACCAA
	R-T 7:TAATACGACTCACTATAGGGACCAGAGTTCCTTCCGCCTC

*Primers F and R were used to amplify the cDNA fragment, and the product was used for the second-round PCR with the primers F and R-T7 to generate an anti-sense probe and with primers R and F-T7 to generate a sense probe.*

## Results

### Histological Analysis of the Juvenile Gonads

**FIGURE 1:**
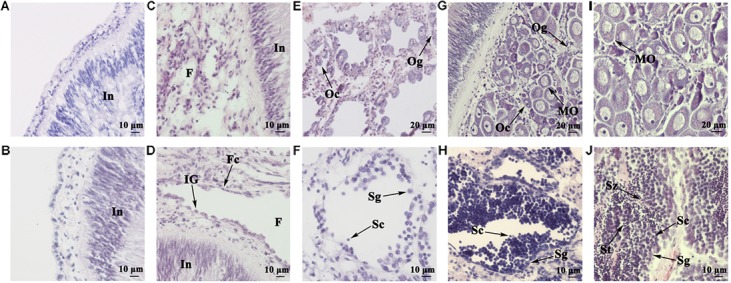
Histological observation of juvenile gonads of different ages. **(A)** Gonad of a 5-month-old juvenile. **(B)** Gonad of a 6-month-old juvenile. **(C)** Gonad of a 7-month-old juvenile. **(D)** Gonad of an 8-month-old juvenile. **(E)** Ovary of a 10-month-old juvenile. **(F)** Testis of a 10-month-old juvenile. **(G)** Ovary of an 11-month-old juvenile. **(H)** Testis of an 11-month-old juvenile. **(I)** Ovary of a 13-month-old juvenile. **(J)** Testis of a 13-month-old juvenile. In, intestine; F, follicle; Fc, follicle cell; IG, indistinguishable gonium; Og, oogonium; Oc, oocyte; MO, mature oocyte; Sg, spermatogonium; Sc, spermatocyte; St, spermatid; Sz, spermatozoon.

Gonads were examined histologically in juveniles aged 5 (shell height ∼10 mm) to 13 months old (shell height ∼60 mm). **Figures 1A,B** show the morphology of 5- and 6-month-old gonads, respectively. As seen, the majority of the gonads are intestine, surrounded by gonadal tissues. In the 7-month-old gonad, some small-sized follicles have formed (**Figure 1C**). In the 8-month-old gonad, the follicles grow bigger but are basically empty, containing some follicle cells and sexually indistinguishable gonia (**Figure 1D**). The 10-month-old gonads are already morphologically differentiated, with oocytes (**Figure 1E**) or spermatocytes (**Figure 1F**) scattering in the follicles. Oogonia, oocytes, and a few mature oocytes coexist in the 11-month-old ovary (**Figure 1G**), and several layers of spermatogonia and spermatocytes are found in the 11-month-old testis (**Figure 1H**), indicating that the gonads are at the growing stage. As shown in **Figures 1I,J**, the 13-month-old gonads have reached full maturity. In the ovary, the follicles are filled with mature oocytes with a polygonal shape due to packing (**Figure 1I**). In the follicles of the testis, diverse germ cells can be detected, including spermatogonia, spermatocytes, spermatids, and spermatozoa (**Figure 1J**).

### Selection of Candidate Genes for Sex Analysis

**FIGURE 2:**
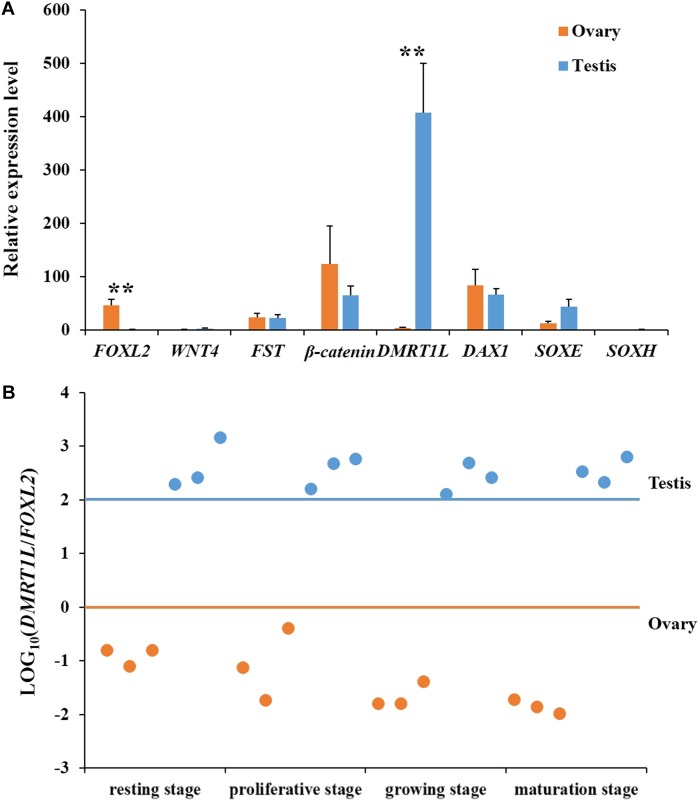
Screening of key genes responsible for scallop sex differentiation. **(A)** Expression profiles of eight candidate genes (*FOXL2*, *WNT4*, *FST*, *β-catenin*, *DMRT1L*, *DAX1*, *SOXE*, and *SOXH*) in scallop ovaries (orange) and testes (blue) at the resting stage. The vertical bars represent the means ± SE (*N* = 7). “^∗∗^” indicates differences that are statistically significant (*P* < 0.01). **(B)** The LOG_10_(*DMRT1L*/*FOXL2*) in ovaries (orange) and testes (blue) at the resting stage, proliferative stage, growing stage, and maturation stage. For each stage, six gonads, including three ovaries and three testes, were assayed. The horizontal lines indicate the threshold for sex differentiation, i.e., orange for ovary and blue for testis, respectively.

To determine the onset of molecular sex differentiation, molecular markers were screened for sex identification. All eight candidate genes (FOXL2, WNT4, FST, β-catenin, DMRT1L, DAX1, SOXE, and SOXH) were identified in the scallop genome. Their expression patterns in ovaries and testes were examined by RT-qPCR. In order to obtain genes that show sexually dimorphic expression in somatic and/or gonia cells and eliminate the effects of gametogenesis, we only used gonads at the resting stage for gene expression analysis (Yu et al., 2017). As shown in **Figure 2A**, only *FOXL2* and *DMRT1L* displayed significant (*P* < 0.01) sexually dimorphic expression, with *FOXL2* being abundant in ovaries, and *DMRT1L* in testes. Therefore, LOG_10_(*DMRT1L*/*FOXL2*) is a potential marker for scallop sex identification, which led us to further investigate its values in the other three reproductive stages (proliferative, growing, and maturation stages). The results also confirmed that LOG_10_(*DMRT1L*/*FOXL2*) could be used to determine the sex of differentiated scallops throughout the reproductive cycle: the value was always lower than 0 for ovaries and higher than 2 for testes (**Figure 2B**).

### Phylogenetic Analyses of FOXL2 and DMRT1L

**FIGURE 3:**
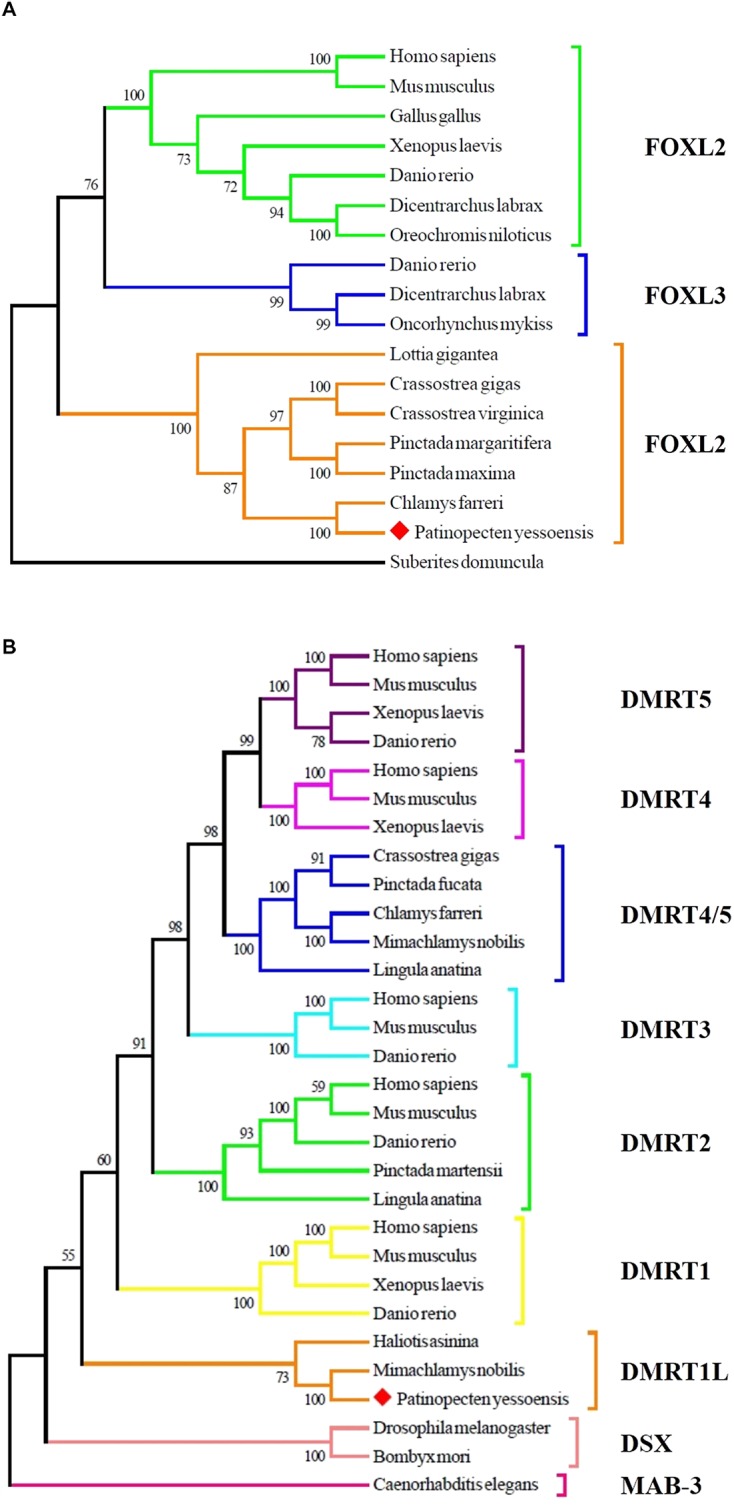
Phylogenetic analyses of FOXL2 **(A)** and DMRT **(B)** proteins. The numbers show the bootstrap percentages (1000 replicates) obtained using neighbor-joining (NJ) method. Different branch colors denote different groups, and red diamonds indicate FOXL2 and DMRT1L of *P. yessoensis*. The protein sequences used for phylogenetic analyses include: FOXL2 of *Homo sapiens* (AAY21823.1), *Mus musculus* (NP_036150.1), *Gallus gallus* (NP_001012630.1), *Xenopus laevis* (BAG69484.1), *Danio rerio* (AAI16586.1), *Dicentrarchus labrax* (AGS36082.1), *Oreochromis niloticus* (AAT36328.1), *Lottia gigantea* (BAQ19215.1), *Crassostrea gigas* (ACN80999.1), *Crassostrea virginica* (XP_022345405.1), *Pinctada margaritifera* (AIE16098.1), *Pinctada maxima* (ATJ00808.1), *Chlamys farreri* (AFB35647.1), *Suberites domuncula* (CAE51212.1); FOXL3 of *Danio rerio* (AAI62838.1), *Dicentrarchus labrax* (AFV13295.1), *Oncorhynchus mykiss* (NP_001117956.1); MAB-3 of *Caenorhabditis elegans* (NP_871909.1); DSX of *Drosophila melanogaster* (NP_731197.1), *Bombyx mori* (AGS48306.1); DMRT1 of *Homo sapiens* (NP_068770.2), *Mus musculus* (NP_056641.2), *Xenopus laevis* (BAE45870.1), *Danio rerio* (AAU04562.1); DMRT1L of *Haliotis asinina* (ACC94178.1), *Mimachlamys nobilis* (AHW85419.1); DMRT2 of *Homo sapiens* (AAD40475.1), *Mus musculus* (NP_665830.1), *Danio rerio* (NP_571027.1), *Pinctada martensii* (ADD97887.1), *Lingula anatina* (XP_013397304.1); DMRT3 of *Homo sapiens* (NP_067063.1), *Mus musculus* (NP_796334.2), *Danio rerio* (AAU89440.1); DMRT4 of *Homo sapiens* (AAI30436.1), *Mus musculus* (AAN77234.1), *Xenopus laevis* (AAV66322.1); DMRT5 of *Homo sapiens* (Q96SC8.2), *Mus musculus* (AAN10254.1), *Xenopus laevis* (AAI70170.1), *Danio rerio* (AAU85258.1); DMRT4/5 of *Crassostrea gigas* (ABS88697.1), *Pinctada fucata* (AIW04133.1), *Chlamys farreri* (ADK55063.1), *Mimachlamys nobilis* (AHW85420.1), *Lingula anatina* (XP_013419494.1).

To determine the identity of the two sexually dimorphic expressed genes, phylogenetic analysis was conducted. As shown in **Figure 3A**, Yesso scallop FOXL2 clustered with FOXL2 from other bivalves and gastropods, and then clustered with vertebrate FOXL2 and FOXL3. In **Figure 3B**, the selected DMRT proteins were clustered into several major groups. The Yesso scallop DMRT, together with DMRT from *Mimachlamys nobilis* and *Haliotis asinina* forms a new group, namely, DMRT1L.

### Localization of *FOXL2* and *DMRT1L* mRNA

**FIGURE 4:**
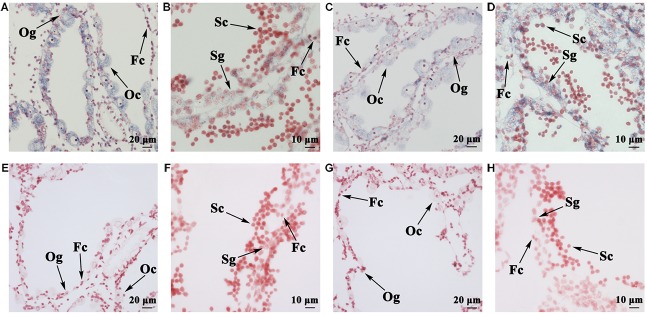
Localization of *FOXL2* and *DMRT1L* in gonads by *in situ* hybridization. **(A)** Localization of *FOXL2* with an anti-sense probe in ovaries. **(B)** Localization of *FOXL2* with an anti-sense probe in testes. **(C)** Localization of *DMRT1L* with an anti-sense probe in ovaries. **(D)** Localization of *DMRT1L* with an anti-sense probe in testes. **(E)** Localization of *FOXL2* with a sense probe in ovaries. **(F)** Localization of *FOXL2* with a sense probe in testes. **(G)** Localization of *DMRT1L* with a sense probe in ovaries. **(H)** Localization of *DMRT1L* with a sense probe in testes. Fc, follicle cell; Og, oogonium; Oc, oocyte; Sg, spermatogonium; Sc, spermatocyte.

Spatial expression of *FOXL2* and *DMRT1L* was detected in the ovary and testis at the proliferative stage after sex differentiation. For both genes, the signals were present in germ cells and follicle cells. Specifically, the anti-sense probe of *FOXL2* was detected in the cytoplasm of oogonia, oocytes, and follicle cells (**Figure 4A**) and faint in testis (**Figure 4B**). *DMRT1L* transcripts were located clearly in all germ cells of the ovary (**Figure 4C**) and testis (**Figure 4D**), but the signal intensity was higher in the testis than in the ovary. No signal was detected in the ovary or testis with the sense probes (**Figures 4E–H**).

### Determining the Timing of Molecular Sex Differentiation

**FIGURE 5:**
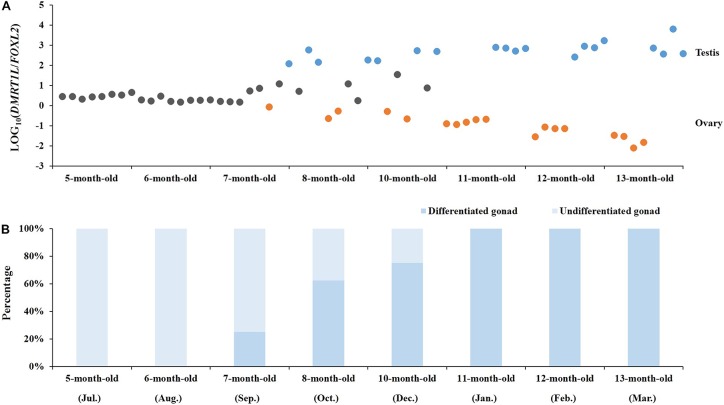
Determination of the timing of molecular sex differentiation in juvenile scallops. **(A)** The dynamic changes in LOG_10_(*DMRT1L*/*FOXL2*) in 5- to 13-month-old juvenile gonads (*N* = 8 for each month). Gray, orange, and blue dots stand for undifferentiated gonads, ovaries and testes, respectively. **(B)** The histogram indicates the corresponding percentage of undifferentiated (light blue) and differentiated gonads (blue) in each month.

The feasibility of LOG_10_(*DMRT1L*/*FOXL2*) for accurate sex identification enables us to determine the timing of gonadal sex differentiation in the Yesso scallop. Here, we traced the dynamic changes of LOG_10_(*DMRT1L*/*FOXL2*) in juveniles aged 5–13 months old. According to **Figure 5A**, LOG_10_(*DMRT1L*/*FOXL2*) values of young individuals (5 and 6 months old) are between 0 and 1, suggesting that these scallops are still undifferentiated. In 7- to 10-month-old scallops, we found a decreasing number of LOG_10_(*DMRT1L*/*FOXL2*) falling within 0–1. This indicates that sex differentiation does not occur simultaneously in all individuals, with some scallops differentiating early when they are 7 months old and some not differentiating until they are 10 months old. However, after reaching 11 months of age, all investigated gonads had differentiated into ovaries or testes. The histogram in **Figure 5B** shows the corresponding differentiation rate for each month based on the LOG_10_(*DMRT1L*/*FOXL2*) values. It reveals an increase in the differentiation rate from 0 in 5- and 6-month-old individuals to 100% in 11- to 13-month-old scallops.

### Coexpression of *FOXL2* and *DMRT1L* in the Juvenile Gonads

**FIGURE 6:**
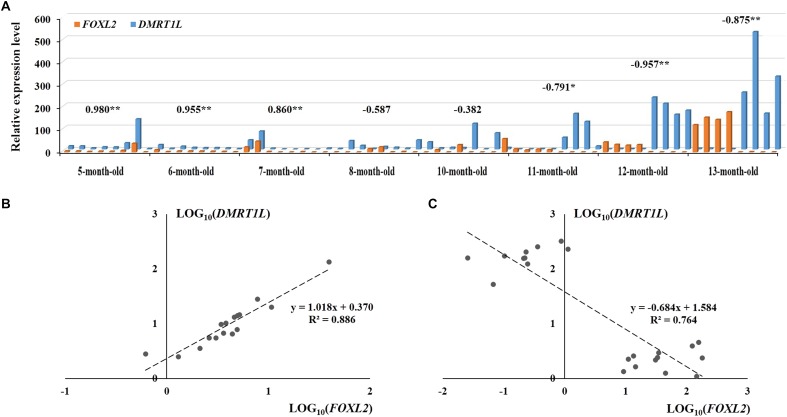
The expression patterns of *FOXL2* and *DMRT1L* in juvenile scallops 5–13 months old. **(A)** The samples correspond to those in **Figure 5A**. The numbers above the bars are the correlation coefficients between LOG_10_(*FOXL2*) and LOG_10_(*DMRT1L*) in the particular months. “^∗^” indicates *P* < 0.05; “^∗∗^” indicates *P* < 0.01. **(B)** The positive correlation between LOG_10_(*FOXL2*) and LOG_10_(*DMRT1L*) for 5- to 6-month-old undifferentiated gonads. **(C)** The negative correlation between LOG_10_(*FOXL2*) and LOG_10_(*DMRT1L*) for 11- to 13-month-old differentiated gonads.

**Figure 6A** shows the temporal expression profiles of both *FOXL2* and *DMRT1L* in juveniles of 5- to 13-month-olds. According to the results, *FOXL2* and *DMRT1L* are relatively low in 5- to 10-month-old scallops, but expression of *FOXL2* in ovaries and *DMRT1L* in testes was dramatically increased in 11- to 13-month-old scallops. During the eight investigated months, there existed two major coexpression patterns between *FOXL2* and *DMRT1L*: (i) a significantly positive correlation for the two undifferentiated stages (*r* = 0.955–0.980, *P* < 0.01), with the regression equation of *y* = 1.018*x* + 0.370 (**Figure 6B**); (ii) significantly negative correlation after the completion of sex differentiation in the last three time points (*r* = −0.791 to −0.957, *P* < 0.05), with the regression equation of *y* = −0.684*x* + 1.584 (**Figure 6C**). Between the undifferentiated and fully differentiated months are three partially differentiated ones, in which correlation coefficients displayed a transition from significantly positive (0.860) to negative (−0.587).

## Discussion

By examining the gonadal expression of eight candidate sex determination or differentiation genes in the resting stage, we found that only two genes (*FOXL2* and *DMRT1L*) displayed sexually dimorphic expression. Both genes are transcription factors, with the former being a forkhead transcription factor and the latter encoding a DM domain-containing protein. Specific sequence information, including conserved domain alignments, has been described previously (Li et al., 2016). According to the transcriptomes of nine adult tissues/organs (Wang et al., 2017), *FOXL2* and *DMRT1L* seem to be specifically expressed in scallop gonads, and no evidence of splice isoforms was found for *DMRT1L* despite that splicing of *DMRT* is common in many animals (Nothiger et al., 1987; Kopp, 2012). Interestingly, sexually dimorphic expression of *FOXL2* (Liu et al., 2012; Teaniniuraitemoana et al., 2014; Zhang et al., 2014) and *DMRT1L* (Shi et al., 2014) occurs not only in the Yesso scallop but also in other bivalves. Therefore, FOXL2 and DMRT1L, like Yin and Yang, may be crucial genes for female and male gonadal differentiation in bivalves. Similarly, in mammals, FOXL2 is an ovarian marker that suppresses genes involved in testicular differentiation from the early embryonic gonad throughout adult life (Cocquet et al., 2002; Ottolenghi et al., 2007; Uhlenhaut et al., 2009; Liu et al., 2014). Although DMRT genes have diverse roles in development and physiology (Huang et al., 2005; Hong et al., 2007; Reitzel et al., 2016), DMRT1 is a deeply conserved gene that is involved in testis differentiation and development in various animal taxa (Naimi et al., 2009b; Kopp, 2012; Matson and Zarkower, 2012; Picard et al., 2015). In mammals, a battle between FOXL2 and DMRT1 for sex control has been proposed (Herpin and Schartl, 2011; Matson et al., 2011). We assume that this battle may be extended to invertebrates, but with different DMRT members.

Spatial expression of *FOXL2* and *DMRT1L* in the ovary and testis reveals that both genes are distributed in germ cells and follicle cells, consistent with previous research in other marine bivalves (Naimi et al., 2009a; Liu et al., 2012). The signal intensity also agrees with the RT-qPCR data. The expression patterns of *FOXL2* and *DMRT1L* make LOG_10_(*DMRT1L*/*FOXL2*) an alternative method to histological examination for scallop sex identification. By tracking LOG_10_(*DMRT1L*/*FOXL2*) at four gonadal developmental stages, we found the value was always lower than 0 for ovaries and higher than 2 for testes, which indicates that in the Yesso scallop, (i) there is no sex reversal once the gonads differentiate, (ii) FOXL2 and DMRT1L may play important roles in both sex maintenance and gametogenesis, and (iii) LOG_10_(*DMRT1L*/*FOXL2*) is an effective solution for sex identification during the resting stage, which is usually indistinguishable by morphology- or histology-based approaches. Since *FOXL2* and *DMRT1L* also show sexually dimorphic expression patterns in other bivalves (Liu et al., 2012; Shi et al., 2014; Teaniniuraitemoana et al., 2014; Zhang et al., 2014), using LOG_10_(*DMRT1L*/*FOXL2*) for sex identification may be applicable in these organisms. For oysters that are capable of sex reversal (Teaniniuraitemoana et al., 2014; Zhang et al., 2014), sex-reversing individuals may be easily screened out by examining the LOG_10_(*DMRT1L*/*FOXL2*) values that fall between the ovary and testis thresholds, which could contribute to a better understanding of the molecular mechanisms underlying sex reversal.

Based on the histological analysis, we found that the developmental process of juvenile gonads can be classified into four stages, namely, undifferentiated, differentiated, growing, and maturation stages, which are consistent with the documentation by Shumway and Parsons (2016). Combined with the dynamic changes of LOG_10_(*DMRT1L*/*FOXL2*) in juvenile scallops, we found that sex differentiation occurs earlier at the molecular level than at the histological level, which is similar to the findings in other organisms (Siegfried, 2010; Haugen et al., 2012; Ayers et al., 2013; Robledo et al., 2015; Tao et al., 2018). In addition, our study shows that although the onset of molecular sex differentiation varies between individuals, it always occurs from 7 to 10 months of age, and almost all of the scallops investigated completed differentiation before 11 months of age. This falls within the time frame of gonadal differentiation documented by Shumway and Parsons (2016) (between 4 months old and 1 year old). It suggests that LOG_10_(*DMRT1L*/*FOXL2*) allows early identification of sex, and is a convenient and accurate way to assess the molecular sex differentiation period. This opens a gate for unraveling the regulatory network of sex differentiation in the Yesso scallop.

The coexpression patterns of *FOXL2* and *DMRT1L* are different before and after gonadal differentiation. Before differentiation, *FOXL2* and *DMRT1L* are relatively low but display unexpected coordinated expression patterns. Combined with the histological results showing that the follicles have not yet formed at this stage, we assume these two genes may coexpress in GSCs that are dispersed in the connective tissues. Sex differentiation co-occurs with the formation of follicles, in which relative expression of *FOXL2* vs *DMRT1L* changes, possibly leading to the differentiation of undifferentiated germ cells into spermatogonia or oogonia. Therefore, we speculate that in Yesso scallops, the undifferentiated gonads directly differentiate into either ovaries or testes. This challenges a previous opinion regarding the existence of sex reversal from male to female through an undifferentiated stage (Maru, 1978). In particular, some researchers claimed that Yesso scallops grow as males at less than one year of age; sex is thereafter reversed to female through an undifferentiated or ambiguous status in half of the scallops, resulting in a sex ratio close to 1:1; after sex reversal, the sex may become stable throughout successive periods of life (Mori et al., 1977; Maru, 1978; Otani et al., 2017). Considering that these studies are generally based on macroscopic observation of gonadal color (testis with white color, ovary with orange or pink color), which could be misleading in some cases, we assume that findings of sex reversal in Yesso scallops may be an artifact. However, since environmental factors have been demonstrated to be involved in the sex determination of many bivalves, we cannot rule out the possibility that the Yesso scallop does undergo sex reversal in Japan.

## Author Contributions

LinZ and SW conceived and designed the experiments. RL, WL, and YZ performed the experiments. YL, MZ, LiaZ, and XH collected the samples. RL and LinZ analyzed the data. RL, LinZ, SW, and ZB wrote the paper. All authors read and approved the final manuscript.

## Conflict of Interest Statement

The authors declare that the research was conducted in the absence of any commercial or financial relationships that could be construed as a potential conflict of interest.

## References

[B1] Ayers K. L. Davidson N. M. Demiyah D. Roeszler K. N. Grützner F. Sinclair A. H. (2013). RNA sequencing reveals sexually dimorphic gene expression before gonadal differentiation in chicken and allows comprehensive annotation of the W-chromosome. *Genome Biol.* 14:R26. 10.1186/gb-2013-14-3-r26 23531366 PMC4053838

[B2] Bull J. J. (1983). *Evolution of Sex Determining Mechanisms.* Menlo Park, CA: Benjamin/Cummings Publishing Company, Inc.

[B3] Cocquet J. Pailhoux E. Jaubert F. Servel N. Xia X. Pannetier M. (2002). Evolution and expression of FOXL2. *J. Med. Genet.* 39 916–921. 10.1136/jmg.39.12.916 12471206 PMC1757225

[B4] Feng Z. Shao M. Sun D. Zhang Z. (2010). Cloning, characterization and expression analysis of Cf-dmrt4-like gene in *Chlamys farreri*. *J. Fish. Sci. China* 17 930–940.

[B5] Goodwin E. B. Ellis R. E. (2002). Turning clustering loops: sex determination in *Caenorhabditis elegans*. *Curr. Biol.* 12 R111–R120. 10.1016/S0960-9822(02)00675-9 11839294

[B6] Haugen T. Almeida F. F. Andersson E. Bogerd J. Male R. Skaar K. S. (2012). Sex differentiation in Atlantic cod (*Gadus morhua* L.): morphological and gene expression studies. *Reprod. Biol. Endocrinol.* 10:47. 10.1186/1477-7827-10-47 22709434 PMC3433390

[B7] Herpin A. Schartl M. (2011). Sex determination: switch and suppress. *Curr. Biol.* 21 R656–R659. 10.1016/j.cub.2011.07.026 21920296

[B8] Heule C. Göppert C. Salzburger W. Böhne A. (2014). Genetics and timing of sex determination in the East African cichlid fish *Astatotilapia burtoni*. *BMC Genet.* 15:140. 10.1186/s12863-014-0140-5 25494637 PMC4278230

[B9] Hong C. S. Park B. Y. Saintjeannet J. P. (2007). The function of Dmrt genes in vertebrate development: it is not just about sex. *Dev. Biol.* 310 1–9. 10.1016/j.ydbio.2007.07.035 17720152

[B10] Huang X. Hong C. S. O’Donnell M. Saintjeannet J. P. (2005). The doublesex-related gene, XDmrt4, is required for neurogenesis in the olfactory system. *Proc. Natl. Acad. Sci. U.S.A.* 102 11349–11354. 10.1073/pnas.0505106102 16061812 PMC1183594

[B11] Kocer A. Reichmann J. Best D. Adams I. R. (2009). Germ cell sex determination in mammals. *Mol. Hum. Reprod.* 15 205–213. 10.1093/molehr/gap008 19218284 PMC2657314

[B12] Kopp A. (2012). Dmrt genes in the development and evolution of sexual dimorphism. *Trends Genet.* 28 175–184. 10.1016/j.tig.2012.02.002 22425532 PMC3350790

[B13] Larkin M. A. Blackshields G. Brown N. P. Chenna R. M. Mcgettigan P. A. Mcwilliam H. (2007). Clustal W. clustal X version 2.0. *Bioinformatics* 23 2947–2948. 10.1093/bioinformatics/btm404 17846036

[B14] Li H. Liu J. Huang X. Wang D. Zhang Z. (2014a). Characterization, expression and function analysis of DAX1 gene of scallop (*Chlamys farreri* Jones and Preston 1904) during its gametogenesis. *J. Ocean Univ. China* 13 696–704. 10.1007/s11802-014-2299-9

[B15] Li H. Zhang Z. Bi Y. Yang D. Zhang L. Liu J. (2014b). Expression characteristics of β-catenin in scallop Chlamys farreri gonads and its role as a potential upstream gene of Dax1 through canonical Wnt signalling pathway regulating the spermatogenesis. *PLoS One* 9:e115917. 10.1371/journal.pone.0115917 25549092 PMC4280107

[B16] Li H. Liu J. Liu X. Zhang Z. (2013). Molecular cloning and expression analysis of wnt4 cDNA from the Zhikong scallop *Chlamys farreri*. *J. Fish. Sci. China* 20 260–268.

[B17] Li Y. Zhang L. Sun Y. Ma X. Wang J. Li R. (2016). Transcriptome sequencing and comparative analysis of ovary and testis identifies potential key sex-related genes and pathways in scallop patinopecten yessoensis. *Mar. Biotechnol.* 18 453–465. 10.1007/s10126-016-9706-8 27234819

[B18] Liu X. L. Li Y. Liu J. G. Cui L. B. Zhang Z. F. (2014). Gonadogenesis in scallop *Chlamys farreri* and Cf-foxl2 expression pattern during gonadal sex differentiation. *Aquac. Res.* 47 1605–1611. 10.1111/are.12621

[B19] Liu X.-L. Zhang Z.-F. Shao M.-Y. Liu J.-G. Muhammad F. (2012). Sexually dimorphic expression of foxl2 during gametogenesis in scallop *Chlamys farreri*, conserved with vertebrates. *Dev. Genes Evol.* 222 279–286. 10.1007/s00427-012-0410-z 22752442

[B20] Maru K. (1978). Studies on the reproduction of a scallop, *Patinopecten yessoensis* (Jay), 2: gonad development in 1-year-old scallops. *Sci. Rep. Hokkaido Fish. Exp. Stn.* 20 13–26.

[B21] Matson C. K. Murphy M. W. Sarver A. L. Griswold M. D. Bardwell V. J. Zarkower D. (2011). DMRT1 prevents female reprogramming in the postnatal mammalian testis. *Nature* 476 101–104. 10.1038/nature10239 21775990 PMC3150961

[B22] Matson C. K. Zarkower D. (2012). Sex and the singular DM domain: insights into sexual regulation, evolution and plasticity. *Nat. Rev. Genet.* 13 163–174. 10.1038/nrg3161 22310892 PMC3595575

[B23] Mori K. Sato R. Osanai K. (1977). Seasonal gonad changes in scallops under culture in Toni Bay, Iwate Prefecture [Japan]. *Bull. Jpn. Soc. Sci. Fish.* 43 1–8. 10.2331/suisan.43.1

[B24] Nagasawa K. Oouchi H. Itoh N. Takahashi K. G. Osada M. (2015). In Vivo administration of scallop gnrh-like peptide influences on gonad development in the yesso Scallop, *Patinopecten yessoensis*. *PLoS One* 10:e0129571. 10.1371/journal.pone.0129571 26030928 PMC4451010

[B25] Naimi A. Martinez A.-S. Specq M.-L. Diss B. Mathieu M. Sourdaine P. (2009a). Molecular cloning and gene expression of Cg-Foxl2 during the development and the adult gametogenetic cycle in the oyster *Crassostrea gigas*. *Comp. Biochem. Physiol. B Biochem. Mol. Biol.* 154 134–142. 10.1016/j.cbpb.2009.05.011 19481171

[B26] Naimi A. Martinez A.-S. Specq M.-L. Mrac A. Diss B. Mathieu M. (2009b). Identification and expression of a factor of the DM family in the oyster *Crassostrea gigas*. *Comp. Biochem. Physiol. A Mol. Integr. Physiol.* 152 189–196. 10.1016/j.cbpa.2008.09.019 18854223

[B27] Ni J. Zeng Z. Han G. Huang H. Ke C. (2012). Cloning and characterization of the follistatin gene from *Crassostrea angulata* and its expression during the reproductive cycle. *Comp. Biochem. Physiol. B Biochem. Mol. Biol.* 163 246–253. 10.1016/j.cbpb.2012.06.006 22771889

[B28] Nothiger R. Leuthold M. Andersen N. Gerschwiler P. Gruter A. Keller W. (1987). Genetic and developmental analysis of the sex-determining gene ‘double sex’ (dsx) of *Drosophila melanogaster*. *Genet. Res.* 50 113–123. 10.1017/S001667230002351X

[B29] Osada M. Harata M. Kishida M. Kijima A. (2004). Molecular cloning and expression analysis of vitellogenin in scallop, *Patinopecten yessoensis* (*Bivalvia, mollusca*). *Mol. Reprod. Dev.* 67 273–281. 10.1016/j.dci.2014.12.004 14735488

[B30] Otani A. Nakajima T. Okumura T. Fujii S. Tomooka Y. (2017). Sex reversal and analyses of possible involvement of sex steroids in scallop gonadal development in newly established organ-culture systems. *Zoolog. Sci.* 34 86–92. 10.2108/zs160070 28397607

[B31] Ottolenghi C. Pelosi E. Tran J. Colombino M. Douglass E. Nedorezov T. (2007). Loss of Wnt4 and Foxl2 leads to female-to-male sex reversal extending to germ cells. *Hum. Mol. Genet.* 16 2795–2804. 10.1093/hmg/ddm235 17728319

[B32] Patnaik B. B. Wang T. H. Kang S. W. Hwang H.-J. Park S. Y. Park E. B. (2016). Sequencing, de novo assembly, and annotation of the transcriptome of the endangered freshwater pearl bivalve, *Cristaria plicata*, provides novel insights into functional genes and marker discovery. *PLoS One* 11:e0148622. 10.1371/journal.pone.0148622 26872384 PMC4752248

[B33] Penman D. J. Piferrer F. (2008). Fish gonadogenesis. Part I: genetic and environmental mechanisms of sex determination. *Rev. Fish. Sci.* 16 16–34. 10.1080/10641260802324610

[B34] Picard M. A. L. Cosseau C. Mouahid G. Duval D. Grunau C. Toulza È (2015). The roles of *Dmrt* (double sex/male-abnormal-3 related transcription factor) genes in sex determination and differentiation mechanisms: ubiquity and diversity across the animal kingdom. *C. R. Biol.* 338 451–462. 10.1016/j.crvi.2015.04.010 26043799

[B35] Piferrer F. Guiguen Y. (2008). Fish gonadogenesis. Part II: molecular biology and genomics of sex differentiation. *Rev. Fish. Sci.* 16 35–55. 10.1080/10641260802324644

[B36] Reitzel A. M. Pang K. Martindale M. Q. (2016). Developmental expression of “germline”- and “sex determination”-related genes in the ctenophore *Mnemiopsis leidyi*. *Evodevo* 7:17. 10.1186/s13227-016-0051-9 27489613 PMC4971632

[B37] Robledo D. Ribas L. Cal R. Sánchez L. Piferrer F. Martínez P. (2015). Gene expression analysis at the onset of sex differentiation in turbot (*Scophthalmus maximus*). *BMC Genomics* 16:973. 10.1186/s12864-015-2142-8 26581195 PMC4652359

[B38] Santerre C. Sourdaine P. Adeline B. Martinez A.-S. (2014). Cg-SoxE and Cg-β-catenin, two new potential actors of the sex-determining pathway in a hermaphrodite lophotrochozoan, the Pacific oyster *Crassostrea gigas*. *Comp. Biochem. Physiol. A Mol. Integr. Physiol.* 167 68–76. 10.1016/j.cbpa.2013.09.018 24120488

[B39] Santerre C. Sourdaine P. Marc N. Mingant C. Robert R. Martinez A.-S. (2013). Oyster sex determination is influenced by temperature—first clues in spat during first gonadic differentiation and gametogenesis. *Comp. Biochem. Physiol. A Mol. Integr. Physiol.* 165 61–69. 10.1016/j.cbpa.2013.02.007 23416889

[B40] Shi Y. Wang Q. He M. (2014). Molecular identification of dmrt2 and dmrt5 and effect of sex steroids on their expressions in *Chlamys nobilis*. *Aquaculture* 426 21–30. 10.1016/j.aquaculture.2014.01.021

[B41] Shumway S. E. Parsons G. J. (2016). *Scallops: Biology, Ecology, Aquaculture, and Fisheries.* New York, NY: Elsevier.

[B42] Siegfried K. R. (2010). In search of determinants: gene expression during gonadal sex differentiation. *J. Fish Biol.* 76 1879–1902. 10.1111/j.1095-8649.2010.02594.x 20557645

[B43] Smith C. A. Roeszler K. N. Ohnesorg T. Cummins D. M. Farlie P. G. Doran T. J. (2009). The avian Z-linked gene DMRT1 is required for male sex determination in the chicken. *Nature* 461 267–271. 10.1038/nature08298 19710650

[B44] Swain A. Lovell-Badge R. (1999). Mammalian sex determination: a molecular drama. *Genes Dev.* 13 755–767. 10.1101/gad.13.7.755 10197976

[B45] Tamura K. Stecher G. Peterson D. Filipski A. Kumar S. (2013). MEGA6: molecular evolutionary genetics analysis version 6.0. *Mol. Biol. Evol.* 30 2725–2729. 10.1093/molbev/mst197 24132122 PMC3840312

[B46] Tanabe T. Yuan Y. Nakamura S. Itoh N. Takahashi K. G. Osada M. (2010). The role in spawning of a putative serotonin receptor isolated from the germ and ciliary cells of the gonoduct in the gonad of the Japanese scallop, *Patinopecten yessoensis*. *Gen. Comp. Endocrinol.* 166 620–627. 10.1016/j.ygcen.2010.01.014 20100484

[B47] Tao W. Chen J. Tan D. Yang J. Sun L. Wei J. (2018). Transcriptome display during tilapia sex determination and differentiation as revealed by RNA-Seq analysis. *BMC Genomics* 19:363. 10.1186/s12864-018-4756-0 29764377 PMC5952695

[B48] Teaniniuraitemoana V. Huvet A. Levy P. Gaertner-Mazouni N. Gueguen Y. Le Moullac G. (2015). Molecular signatures discriminating the male and the female sexual pathways in the pearl oyster *Pinctada margaritifera.* *PLoS One* 10:e0122819. 10.1371/journal.pone.0122819 25815473 PMC4376701

[B49] Teaniniuraitemoana V. Huvet A. Levy P. Klopp C. Lhuillier E. Gaertner-Mazouni N. (2014). Gonad transcriptome analysis of pearl oyster *Pinctada margaritifera*: identification of potential sex differentiation and sex determining genes. *BMC Genomics* 15:491. 10.1186/1471-2164-15-491 24942841 PMC4082630

[B50] Tong Y. Zhang Y. Huang J. Xiao S. Zhang Y. Li J. (2015). Transcriptomics analysis of *Crassostrea hongkongensis* for the discovery of reproduction-related genes. *PLoS One* 10:e0134280. 10.1371/journal.pone.0134280 26258576 PMC4530894

[B51] Uhlenhaut N. H. Jakob S. Anlag K. Eisenberger T. Sekido R. Kress J. (2009). Somatic sex reprogramming of adult ovaries to testes by FOXL2 ablation. *Cell* 139 1130–1142. 10.1016/j.cell.2009.11.021 20005806

[B52] Wang S. Zhang J. Jiao W. Li J. Xun X. Sun Y. (2017). Scallop genome provides insights into evolution of bilaterian karyotype and development. *Nat. Ecol. Evol.* 1:0120. 10.1038/s41559-017-0120 28812685 PMC10970998

[B53] Yang M. Xu F. Liu J. (2015). Molecular cloning and expression of Wnt4 gene in pacific oyster *Crassostrea gigas*. *Oceanol. Limnol. Sin.* 46 35–42.

[B54] Yu F.-F. Wang M.-F. Zhou L. Gui J.-F. Yu X.-Y. (2011). Molecular cloning and expression characterization of Dmrt2 in Akoya pearl oysters, *Pinctada martensii*. *J. Shellfish Res.* 30 247–254. 10.2983/035.030.0208

[B55] Yu J. Zhang L. Li Y. Li R. Zhang M. Li W. (2017). Genome-wide identification and expression profiling of the SOX gene family in a bivalve mollusc *Patinopecten yessoensis*. *Gene* 627 530–537. 10.1016/j.gene.2017.07.013 28694209

[B56] Zarkower D. (2001). Establishing sexual dimorphism: conservation amidst diversity? *Nat. Rev. Genet.* 2 175–185. 10.1038/35056032 11256069

[B57] Zhang N. Xu F. Guo X. (2014). Genomic analysis of the Pacific oyster (*Crassostrea gigas*) reveals possible conservation of vertebrate sex determination in a mollusc. *G3* 4 2207–2217. 10.1534/g3.114.013904 25213692 PMC4232546

