# Supplementary material for: FOXL2 and DMRT1L Are Yin and Yang Genes for Determining Timing of Sex Differentiation in the Bivalve Mollusk Patinopecten yessoensis
Source: Front Physiol. 2018 Aug 22;9:1166. doi: 10.3389/fphys.2018.01166 (PMC6113668; doi:10.3389/fphys.2018.01166)
Supplement: Supplementary file 1 [file Data_Sheet_1.ZIP › Supplementary material/Supplementary material.pdf]

FOXL2 and DMRT1L are Yin and Yang genes for determining timing of sex differentiation in the bivalve mollusk *Patinopecten yessoensis*

Ruojiao Li [1, 2+], Lingling Zhang [1, 3+*], Wanru Li [1], Yang Zhang [1], Yangping Li [1], Meiwei Zhang [1], Liang Zhao [1], Xiaoli Hu [1, 3], Shi Wang [1, 2] and Zhenmin Bao [1, 3]

[1] MOE Key Laboratory of Marine Genetics and Breeding, Ocean University of China, Qingdao, China
[2] Laboratory for Marine Biology and Biotechnology, Qingdao National Laboratory for Marine Science and Technology, Qingdao, China
[3] Laboratory for Marine Fisheries Science and Food Production Processes, Qingdao National Laboratory for Marine Science and Technology, Qingdao, China

[+]These authors contributed equally to this work.

***Correspondence**
Dr. Lingling Zhang
lingling80@ouc.edu.cn

Supplemental material

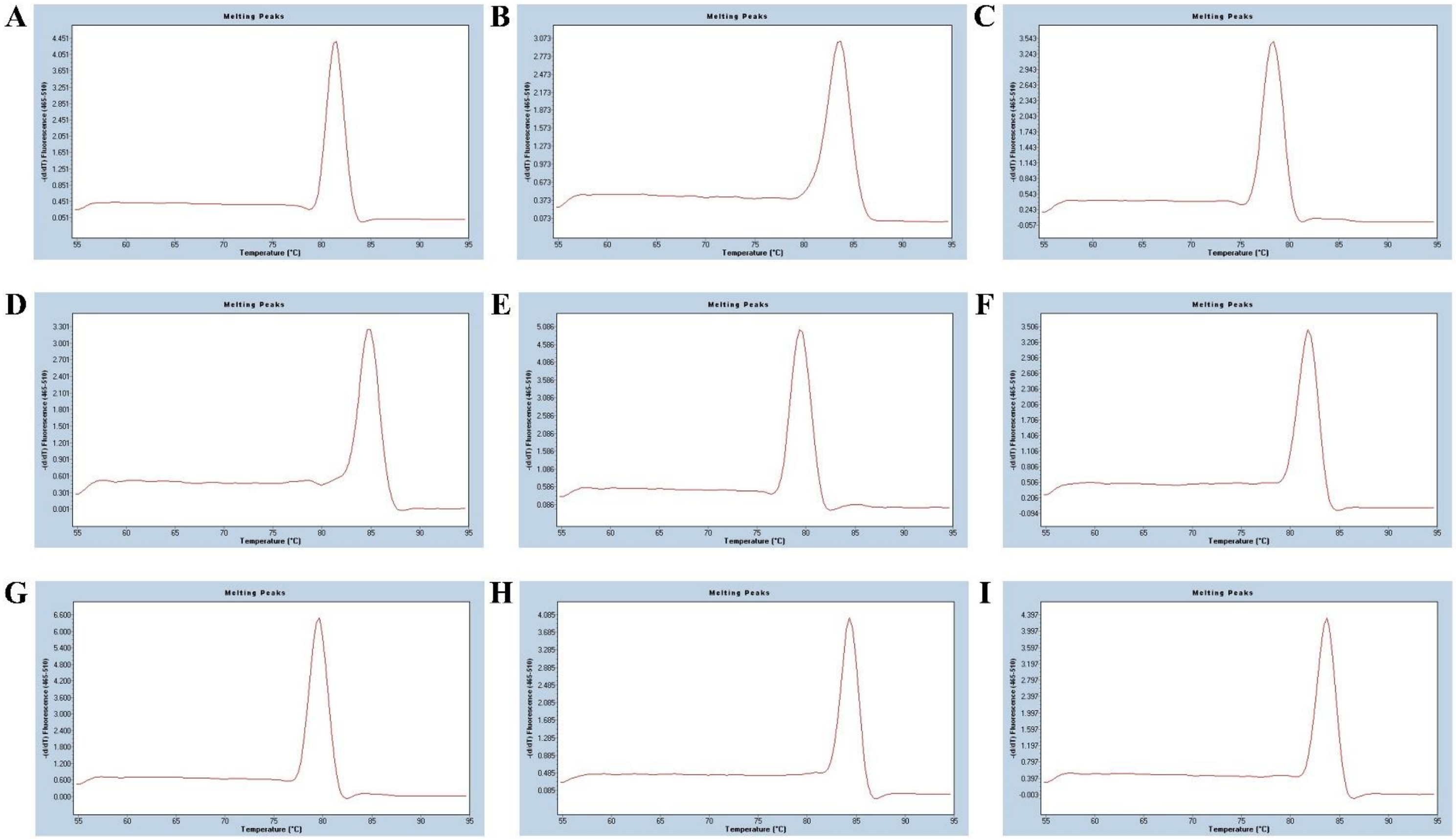

Supplementary Figure 1. Melting curves of internal reference gene and candidate sex determination/differentiation genes. (**A**) *EF1A*; (**B**) *FOXL2*; (**C**) *WNT4*; (**D**) *FST*; (**E**) *β-catenin*; (**F**) *DMRT1L*; (**G**) *DAX1*; (**H**) *SOXE*; (**I**) *SOXH*.
